# Electrospinning of Chitosan-Based Solutions for Tissue Engineering and Regenerative Medicine

**DOI:** 10.3390/ijms19020407

**Published:** 2018-01-30

**Authors:** Saad B. Qasim, Muhammad S. Zafar, Shariq Najeeb, Zohaib Khurshid, Altaf H. Shah, Shehriar Husain, Ihtesham Ur Rehman

**Affiliations:** 1 Department of Restorative and Prosthetic Dental Sciences, College of Dentistry, Dar Al Uloom University, P.O. Box 45142, Riyadh 11512, Saudi Arabia; s.qasim@dau.edu.sa; 2 Department of Restorative Dentistry, College of Dentistry, Taibah University, Al Madinah, Al Munawwarah 41311, Saudi Arabia; 3 Department of Dental Materials, Islamic International Dental College, Riphah International University, Islamabad 44000, Pakistan; 4 Restorative Dental Sciences, Al-Farabi Colleges, Riyadh 361724, Saudi Arabia; shariqnajeeb@gmail.com; 5 College of Dentistry, King Faisal University, P.O. Box 380, Al-Hofuf, Al-Ahsa 31982, Saudi Arabia; drzohaibkhurshid@gmail.com; 6 Department of Preventive Dental Sciences, College of Dentistry, Dar Al Uloom University, Riyadh 11512, Saudi Arabia; a.shah@dau.edu.sa; 7 Department of Dental Materials, College of Dentistry, Jinnah Sindh Medical University, Karachi 75110, Pakistan; shehriarhusain@gmail.com; 8 Materials Science and Engineering Department, Kroto Research Institute, University of Sheffield, Sheffield S3 7HQ, UK; i.u.rehman@sheffield.ac.uk

**Keywords:** chitosan, composite solutions, electrospinning, regeneration, tissue engineering

## Abstract

Electrospinning has been used for decades to generate nano-fibres via an electrically charged jet of polymer solution. This process is established on a spinning technique, using electrostatic forces to produce fine fibres from polymer solutions. Amongst, the electrospinning of available biopolymers (silk, cellulose, collagen, gelatine and hyaluronic acid), chitosan (CH) has shown a favourable outcome for tissue regeneration applications. The aim of the current review is to assess the current literature about electrospinning chitosan and its composite formulations for creating fibres in combination with other natural polymers to be employed in tissue engineering. In addition, various polymers blended with chitosan for electrospinning have been discussed in terms of their potential biomedical applications. The review shows that evidence exists in support of the favourable properties and biocompatibility of chitosan electrospun composite biomaterials for a range of applications. However, further research and in vivo studies are required to translate these materials from the laboratory to clinical applications.

## 1. Introduction

Electrospinning (ES) is a process that utilises an electric field to control the deposition of polymer fibres onto target substrates. Originally, the process was devised for the textile industry [1]. It is now lauded for its capabilities to economically and efficiently fabricate non-woven meshes of fibres specifically in the field of tissue engineering (TE) where these fibrous scaffolds can mimic both the natural form and function of the extracellular matrix (ECM) [2,3,4]. Historically, ES was first observed by Rayleigh in 1897, and was further explored by Zeleny in 1914. Later in 1934, Formhals patented the process of ES for the production of collagen acetate fibres using a strong voltage of 57 kV [5]. Since 1980s, ES has been used to create submicron to nano-meters (nm) sized fibres. Fibres of varying characteristics can be acquired by altering different processing parameters. Currently, the ES is not being used solely in the healthcare industry [6,7], but also has a wide range of applications in other fields including energy [8], waste water [9], textile [10] and security domains [6,11,12,13]. According to the European Patent Office, until 2013, more than 1891 patents had been filed using the term “ES” and 2960 with the term “nano-fibres” in title and abstract [1,14]. More than 200 institutions and universities worldwide have explored ES as a means of producing different types of nano-fibres [5,7]. In terms of dental applications of ES, more than 45 scientific research papers have been published since 2005 [7].

ES has been adapted to obtain natural and synthetic polymer fibrous mats that mimic the extra cellular matrix (ECM). Amongst available biopolymers, CH and its naturally derived composites have been widely adapted for TE applications. Its versatility with respect to variations in the molecular weights and degree of deacetylation in order to target a clinical condition in the field of regenerative medicine is adapted very efficiently. For ease of understanding, there is a brief description of the ES process, various parameters affecting the fabrication and properties of electrospun nano-fibres. The aim of the current review is to assess the current literature about the electrospinning of chitosan and its composites with other natural polymers to be utilized in TE and regenerative applications.

## 2. Solution Electrospinning Process

**Figure 1:**
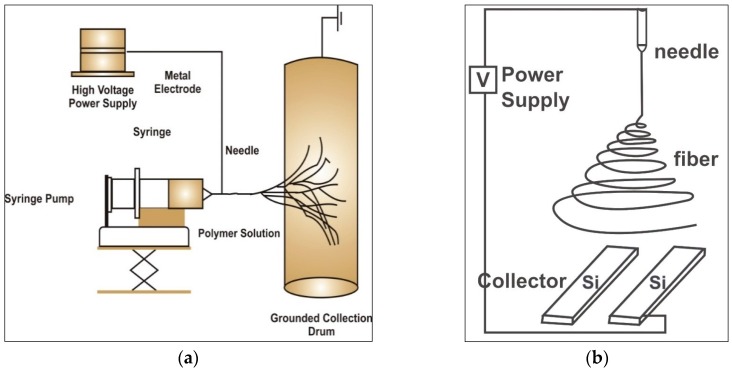
The electrospinning process shown schematically (**a**) electrospinning equipment plate or rotating mandrel (**b**) aligned collection plates for electrospun nano-fibres [7].

The process of ES is based on obtaining fine fibres from polymer solutions via electrostatic forces. The electrospun fibres have small diameters and significantly larger surface area compared to conventional fibres. The essential components of ES are: 1-high voltage power supply, 2-spinneret and a grounded collecting plate such as a metallic screen, and 3-plate or rotating mandrel (Figure 1).

A direct current (DC) voltage is used to generate a potential difference between two terminals within the range of 1–30 kVs that injects a charge of a certain polarity into the polymer solution to accelerate a jet towards a collector of opposite polarity [15]. A syringe pump is used to pump the completely dissolved polymer solutions into the metallic capillary tube [16,17]. The ES equipment can be setup in vertical or horizontal orientations (Figure 1a,b).

In brief, a polymer solution is held by its surface tension at the capillary tip that is subjected to the potential difference created between the spinneret and anode surface (collector). Due to this electric field, a charge is triggered on the liquid surface, changing the pendant drop to a falling jet. When this field reaches a critical value, the repulsive electric forces overcome the surface tension forces. Finally, an unstable charged jet of polymer solution is extruded from the tip of the Taylor cone (conical profile of solvent which bends stretches and thins). Consequently, an unstable and a rapid whipping jet ensues between the capillary tip and collector. The resultant evaporation of the solvent leaves the polymer nano-fibres behind on the collector [18,19].

### 2.1. Processing Parameters

The characteristics of electrospun fibres are governed by various factors related to the ES dope such as concentration, molecular weight, surface tension, viscosity and conductivity/surface charge density of the polymer solution. In addition, equipment factors such as voltage, feed or flow rate of polymer loaded in the syringe, type of collector and the distance between the collector and needle tip can affect the morphology of electrospun fibres [7]. For instance, the shape of the polymer solution drop is affected by the applied voltage, viscosity of the solution and the flow rate of the syringe [20]. It has also been reported that the higher electrostatic forces cause more stretching of solution due to columbic forces in the jet, thereby generating a stronger electric field ultimately reducing the fibre diameter and higher rate of evaporation of solvent [16,21]. On the other hand, a few researchers have observed that the fibre diameter is proportional to the voltage [22,23]. Velocity of the jet and the material transfer rate are affected by the rate at which the polymer solution is pushed out of a syringe [24]. Slower feed rate facilitates enough time for evaporation of the solvent and is likely to reduce the fibres’ diameter at the expense of prolonged processing time. The flow rate affects the pores and surface area as well. Fong et al. have reported the fabrication and detailed characterization of electrospun beaded fibres and suggested that parameters such as charge density, solution viscosity and surface tension are the main factors for controlling bead formation [23].

In terms of collectors, several materials can be used to collect the electrospun nano-fibres [25,26]. Aluminium foils are the most commonly used collectors since, the material must be conductive and remain isolated from the axel [27]. Other alternate options included conductive paper/cloth, parallel or guided bar, wire mesh, rotating wheel or rods. Wang et al. [25] studied the difference in the characteristics of fibres collected on an aluminium foil and a wire screen. They observed that due to the different conducting areas on the wire screen, there was more bead formation compared to an aluminium foil. Fibre alignment and diameter can be changed by altering the speed of a rotating collector. High speed rotating collectors tend to produce more aligned and narrower fibres compared to collectors rotating at a slower rate [28]. Further modification of collectors using different attachments such as pins, [29] mandrels [26] and rods [30] can be achieved to collect electrospun fibres. In order to control fibre characteristics and diameter, the tip to collector distance is also a critical factor. According to Buchko et al. a larger distance between the tip and the collector produces round fibres while a shorter distance produces flat fibres [31]. Reducing the tip to collector distance does not allow sufficient time for the evaporation of the ES solvent prior to hitting the collector. Hence, the moist/wet fibres hitting the collector change their morphology to flat. Hence, the optimal distance is essential to facilitate the evaporation of solvent from the nano-fibres prior to hitting the collector [16].

### 2.2. Solution Parameters

Solution parameters such as concentration and viscosity influence the electrospun fibre morphology. For instance, lower concentrations result in finer fibres and increased number of beads, while concentrated solutions result in thicker bead free fibres or a reduced number of beads [32]. As the concentration of solution increases, fibres form a more spindle like structure and display uniformly thicker fibres due to higher viscosity [21,33]. Highly viscous solutions can be electrospun to form fibres because the flow rate is unable to be maintained at the tip of the solution which leads to formation of large fibres [33]. As the viscosity is directly related to the concentration, each polymer solution has an optimal concentration for ES.

Solution viscosity is another vital parameter that affects the electrospun fibres in terms of size and morphology. ES of uniform bead-free nano-fibres requires the polymer solution to have an optimal viscosity. Very thick fibres can result if the viscosity is high enough to hinder the ejection of the polymer solution. At a viscosity lower than optimal, the continuous fibre formation is less likely and results in droplets or excessive bead formation. The viscous polymers result in extending the stress relaxation times and limiting the breakage of polymer jets. Consequently, fibres acquired at relatively higher viscosity showed more uniform fibres. Similarly, low surface tension of the solvent facilitates production of nano-fibres with less or no beads [23]. Polymer solutions with greater surface tension obscure ES because of jet instability and generation of sprayed droplets [5,19,34]. Charge density is another important factor defining the outcome of the fibres. Narrower diameter fibres can be obtained if electrical conductivity is increased. In contrast, if the solvent is less conductive this results in insufficient elongation of the jet by electrical force producing uniform fibres and bead formation. The majority of the polymers are usually conductive. The electrospinning polymer solutions have charged ions that are highly efficient in jet formation [16].

The molecular weight (Mw) affects the solution properties such as dielectric constant, conductivity, electrical, viscosity, surface tension, and rheological properties [7,16]. The high Mw solutions are opted corresponding to the intended viscosity for generating fibres. The Mw determines the extent and number of polymer chain entanglements in the solution [5,18,22]. 

### 2.3. Effect of Nanoinclusions on CH (Chitosan) Electrospinning

Nanoparticles of various natural and synthetically derived materials have a significant impact on the process of electrospinning [35]. Additions of various natural or synthetic nano biomaterials affects the electrospun fibre morphology, size and diameter [36,37]. The addition of nano hydroxyapatite (HAp) has been explored to obtain fibres within the range of 300 nm. Liverani et al. reported a critical finding about Hap-containing fibres that showed a decrease in average diameter with respect to pure CH fibres [38]. Few other studies have also investigated the effect of nano HAp addition to CH in order to obtain fibres for TE applications. Zhang and co-workers were able to obtain fibres with diameters of 214 nm mimicking naturally mineralized counterparts [39]. In a similar study, Thein-Han et al. compared the addition of micro and nano HAp to CH solutions [40]. Results were suggestive that electrospun fibres obtained whilst using nano HAp had better stem cell attachment compared to micro HAp [40,41].

## 3. Chitosan-Based Polymers for Solution Electrospinning

A wide variety of natural and synthetic polymers and their composite blends have been used for ES. Examples of electrospun synthetic polymers include poly glycolic acid (PGA), poly lactide co-glycolide (PLGA), polycaprolactone (PCL), polyurethane (PU), poly lactic acid (PLA), polystyrene (PS) and poly vinyl alcohol (PVA). Some of polymers available naturally for ES are, silk, cellulose, collagen, gelatine, hyaluronic acid and CH. Natural and synthetic polymers are also used in combination to manipulate the materials properties (such as thermal stability, mechanical strength and barrier properties) depending on the specific application. Other properties such as cellular affinity, morphological, structural, pore size and degradation can also be altered by copolymers [5].

### 3.1. Chitosan

**Figure 2:**
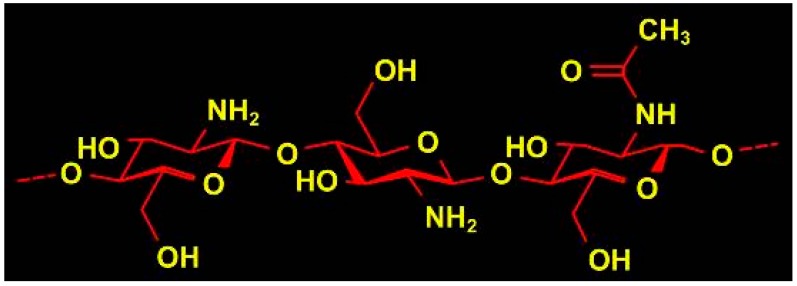
Chemical structure of chitosan showing amide and hydroxyl group that can react and readily form bonds with other natural or synthetic polymers/biomolecules [44].

Chitosan (CH) is a polysaccharide biomaterial composed of (1–4) acetamido 2 deoxy-β-d glucan, (*N*-acetyl d glucosamine and 2 amino 2 deoxy-β-d glucans. The structure of the biopolymer is shown in Figure 2. CH is obtained by partial deacetylation of chitin. The presence of amino groups in the CH structure differentiates CH from chitin. The degree of deacetylation of CH is indicative of the number of amino groups [42].

**Figure 3:**
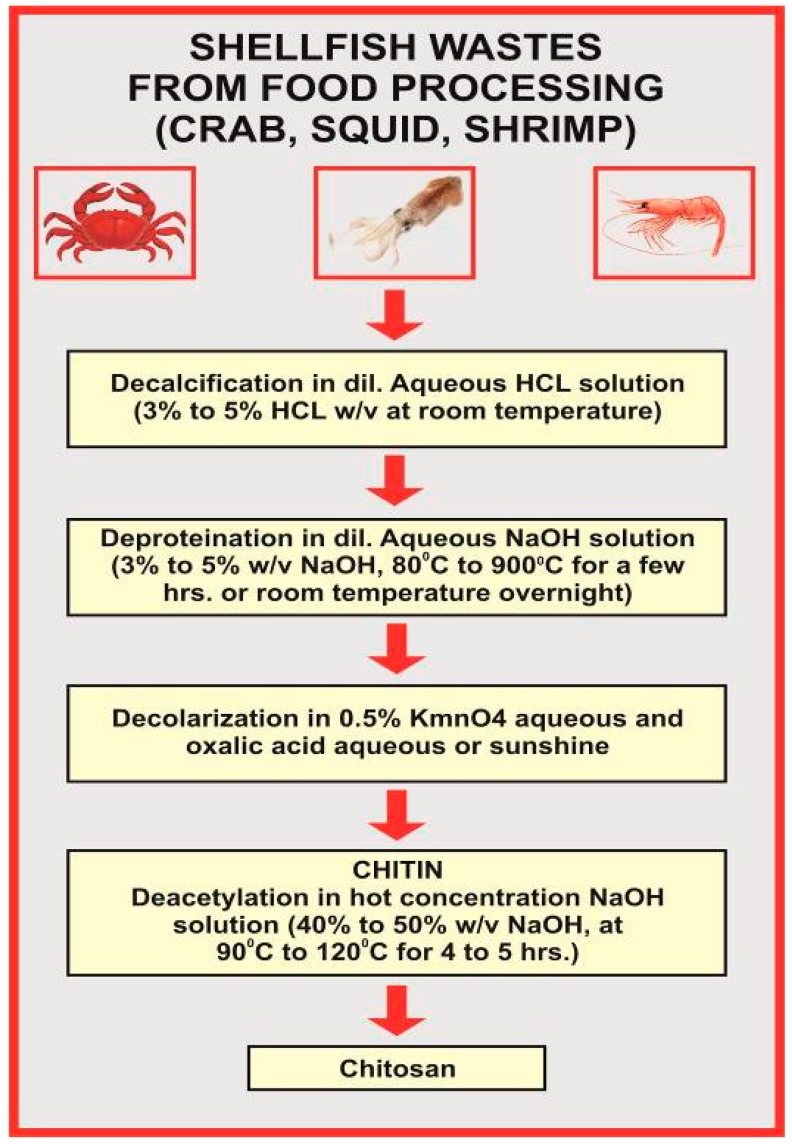
Illustration depicting the deacetylation process adapted to extract CH from chitin [45].

The deacetylation of CH is performed either by chemical hydrolysis under harsh alkaline conditions or enzymatic hydrolysis [42]. The alkali extracts the protein and deacetylated chitin at the same time. The processing of crustacean shells involves the extraction of proteins and the dissolution of calcium carbonates, that is accumulated in the crab shell at high concentrations [43]. The deacetylation of chitin is rarely complete; when the degree of deacetylation above 60%, chitin becomes CH (Figure 3).

Fabrication of electrospun CH micro or nano-fibres is not an easy task. Common solvents used for dissolving CH are triflouroacetic acid (TFA) or composite solutions of diluted acetic acid copolymerized with polyethylene oxide (PEO) [46]. Different customised approaches performed are either neutralization using the alkaline compounds or cross linkers such as gluteraldehyde and genipin. Hence, the neutralization may ultimately lead to partial or complete loss of features [46].

The majority of the acidic solvents can easily solubilise CH. Its protonation changes CH into a polyelectrolyte in acidic solutions. CH is not able to produce continuous fibres due to the consistent formation of droplets. The high electric field during ES results in repulsive forces among ionic groups within the polymer backbone, hence inhibiting the formation of continuous fibres [47,48]. Although the dual needle setup for CH electrospinning has been rarely reported, it results in repelling of fibres. In addition, adjusting the ideal viscosity of CH electrospinning dope is also challenging. Its rigid d-glucosamine structures, high crystallinity and ability to form hydrogen bonding is responsible for the poor solubility in commonly available organic solvents [49].

Being a cationic polymer, CH in aqueous solution has poly-electrolytic effects. The presence of charged groups results in the significant expansion of polymer coils. In case of electrolyte free polymer solution, the polymer coils shrink and concentrate up. The use of very toxic organic solvents such as hexa-fluoro isopropanol (HFIP) or triflouro acetic acid (TFA) denatures the properties and structure of natural CH [50] and further breaks the inter-chain interactions. Although the number of studies reporting CH fibres for various applications is increasing, each year, very little information is available about the obstacles encountered in obtaining desired fibre properties such as diameter and size. Due to the highly crystalline nature of CH, once dissolved in organic solvents, the formation of hydrogen bonds further complicates the spin-ability [51,52]. In order to overcome this issue, a very low concentration of diluted acetic acid and a fibre-forming agent (PEO) (95:5, CH:PEO) can be used. Sun and Li have mentioned about the problems associated with high viscosity CH limiting its ability to form fibres. It was suggested to perform alkali treatment to hydrolyse CH chains and use a lower molecular weight of CH to overcome this problem [53].

**Figure 4:**
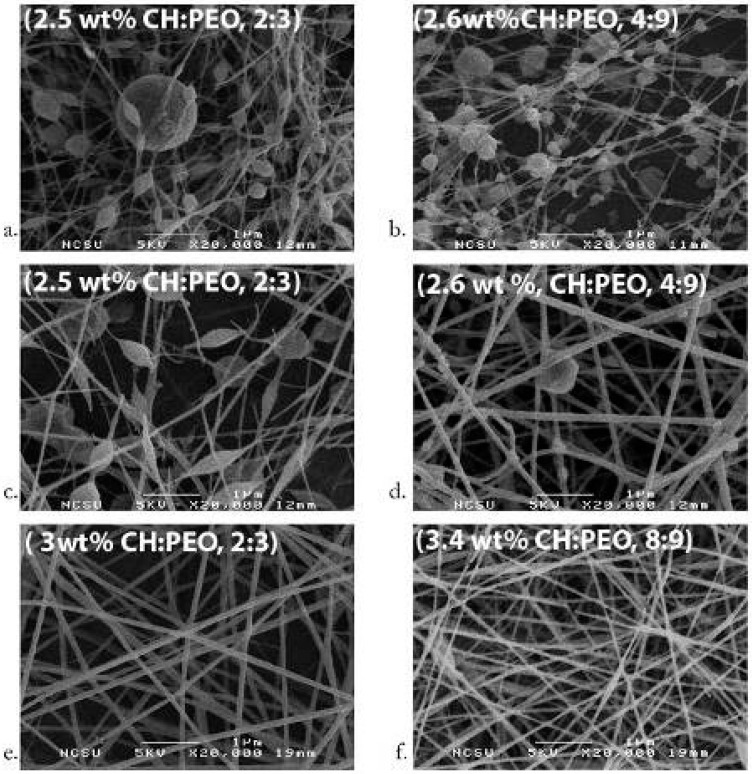
Chitosan PEO nano-fibres depicting the effects of acetic acid concentration, (**a**) 2:3 CH:PEO in 45% (**b**) 4:9 CH:PEO in 36%; (**c**) 2:3 CH:PEO with 2.5 wt.% total polymer 40%; (**d**) 4:9 CH:PEO with 2.6 wt.% total polymer 32%; (**e**) 2:3 CH:PEO with 3 wt.% total polymer blend and 32%; (**f**) 8:9 CH:PEO with 3.4 wt.% total polymer 32% of total acetic acid concentration [59]; scale bar represents 1 μm (Adapted with permission from publisher).

Previously, a number of studies have reported amide bond (NH) formation of CH with organic solvents [54,55]. Yao et al. used lactic acid to generate lactamidated CH in the form of films. CH films were tested for their biocompatibility using fibroblasts [56]. Qu et al. [57] and Toffey et al. [58] used acetic and propionic acids for the regeneration of chitin through amide bond formation. Scanning electron microscopy (SEM) revealed that increase in the polymer concentration resulted in thicker fibres. PEO and CH solutions showed phase separation over time; hence, blended solutions must be electrospun at the earliest preferably within 24 h of blending to prevent any chemical denaturation. The addition of salt before and during ES to these solutions stabilizes them for an extended period of time (Figure 4) [46,59].

### 3.2. Chitin/Silk Composite Nano-fibres

Silks are natural polymers produced by insects such as spiders and silkworms. Natural silk fibres have shown favourable features (biocompatibility, non-toxicity) needed for biomaterial applications [60,61]. In addition, the mechanical properties are excellent [62]. Therefore, it has the ability to function under a range of temperatures and humidity [63]. Silk boasts an extensive track record, spanning a period of decades, as a surgical suture material [64]. Silk has also been explored for various biomaterial applications such as TE scaffolds [65,66], and drug delivery [67,68] and bio-dental applications [69,70,71,72]. There are many silk-producing animal organisms, however, the main source remains silk worms [73].

Silk harvested from the silkworm (*Bombyx mori)* has the advantage of economic importance as can be domesticated in farms [74]. The *Bombyx mori* (BM) silk has two protein components, a water soluble sericin and water insoluble hydrophobic silk fibroin [74,75]. Glue-like sericin is amorphous in nature and is rich in serine (Mw ~ 10–300 kDa). It makes approximately 20–30 wt.% of BM silk [76]. Sericin acts as protective coating of silk filaments and cocoons [77] that is permeable to moisture and resistant to oxidation and UV [78]. The sericin has been reported to be associated with the allergic and immunological reactions [79,80], and is hence important to remove sericin completely from fibroin before any biological application can be considered [75,81,82].

The structural component of BM silk is silk fibroin protein (~75 wt.% of total silk) that is a large macromolecule comprised of ~5000 amino acid units [83,84]. The silk fibroin (SF) has crystalline (~66%) and amorphous (~33%) components [85]. The crystalline SF has repeating amino acid units mainly alanine (A), glycine (G) and serine (S) in a typical sequence [G-A-G-A-G-S]_n_. It forms a β-sheet structure in the spun fibres which is responsible for good mechanical properties [85,86]. In contrast, the amorphous part is mainly composed of phenylalanine (F) and tyrosine (Y). The large side chains of these amino acids lead to hygroscopic properties [87]. SF is further divided into heavy and light chains (H-fibroin and L-fibroin) bonded to each other through disulfide bridges [88,89]. In addition, a glycoprotein (P25) is attached to the SF molecules by non-covalent interactions [89,90]. Considering the unique properties of nanocomposite materials [91] and natural silk, a number of researchers [92,93,94] have electrospun CH/chitin and silk fibroin (SF) blends using various combinations and solvents (Table 1). Park et al. [93] reported the fabrication of electrospun SF/CH composite nano-fibres using formic acid as an ES solvent. Formic acid is an organic solvent that is highly volatile and has been successfully used for silk fibroin ES [71,95]. The average fibre diameter was reduced with a narrow diameter distribution compared to silk-only nano-fibres. The ionic component of CH results in the increased conductivity of the ES solution, hence, a stronger jet. In addition, intermolecular interactions for example, hydrogen bonding between SF and CH solutions may affect the final properties [93]. The SF nano-fibres are treated with alcoholic solution to induce β-sheet conformation that in turn improves the mechanical properties [96,97]. The CH has a rigid backbone, hence accelerating the conformational changes in SF electrospun nano-fibres [93].

Another study [98] reported ES of chitin/SF-blended fibres using 1,1,1,3,3,3-hexafluoro-2-propanol (HFIP) as an ES solvent. The electrospun fibres were characterized for morphology, dimensional stability and cyto-compatibility. Due to the immiscible nature of SF and chitin, electrospun fibres showed phase-separated structures. The average fibre diameter was reduced by increasing the chitin contents. The solvent induced crystallization and improved the dimensional stability of chitin/SF nano-fibres. In addition, biological properties such as biocompatibility, cell attachment and spreading were evaluated. In terms of potential TE scaffold applications, these electrospun blends exhibited promising characteristics including excellent biocompatibility, cell attachment and spreading for epidermal keratinocytes and fibroblasts [94,98]. Similar findings have been reported by Yoo et al. [94]; chitin and SF solutions were electrospun simultaneously using a hybrid ES technique and nano-fibres were collected at a rotating target. The composition of hybrid materials was controlled by adjusting the solution flow rates. The average fibre diameter was decreased with increasing proportions of CH.

Increasing the proportion of SF improved the mechanical properties whereas higher proportions of CH improved the antibacterial activity of the electrospun fibres. Authors suggested that CH/SF electrospun membranes are a promising candidate for wound healing applications. Chen et al. [99] reported the fabrication of bead-free electrospun nano-fibres of CH/SF blends. The composite nano-fibres were characterized for cellular response using human foetal osteoblasts. SF/CH nano-fibres encouraged the proliferation and differentiation of human foetal osteoblasts. Authors reported the suitability of these materials for bone regeneration applications by choosing a suitable composition. Recently, composite nano-fibres [*N*-carboxyethyl CH/PVA/SF] have been electrospun using aqueous solutions with suspended SF nanoparticles [92]. These materials had a benefit of being electrospun from aqueous solutions instead of organic solvents during cytotoxicity testing of mouse fibroblasts (L929). These nanomaterials demonstrated good biocompatibility, and hence, can be considered for potential TE applications such as skin regeneration wound dressings [92]. It is clear from previous studies that CH and silk fibroin are compatible with each other for the purpose of ES. A wide range of proportions coupled with ES techniques have been tested with promising outcomes for tissue regeneration applications. However, all reported studies (Table 1) have been conducted in the laboratory environment focusing mainly on physical, mechanical and biological properties. 

Chitosan/silk fibroin composite materials showed satisfactory properties and biocompatibility essentially required for tissue regeneration and biomedical applications. However, further research including in vivo studies is required to prove these claims for more practical and clinical applications.

### 3.3. Collagen Chitosan (CC)

Collagen is another natural polymer that has excellent properties (such as biodegradability and biocompatibility) and has been widely explored for TE applications [101,102]. Collagen has been electrospun with and without blending with other polymers such as natural silk [57,58,103], PCL [104], PEO [105] and chitosan [44,101,106]. The purpose of blending other materials with collagen is obvious in terms of modifying the final properties according to potential applications. For example, collagen-CH (CC) blends can modify the mechanical and biological properties to mimic the natural extracellular matrix [107,108]. Previous literature [108,109,110] has reported the effects of added CH to properties of collagen-based biomaterials. For instance, addition of CH is known to modify the collagen helical characteristics by introducing additional hydrogen bonding that ultimately changes the physical properties. Fourier Transform Infrared (FTIR) spectroscopic analysis has confirmed such molecular interactions between collagen and CH blends [107]. In terms of fibre morphology of CC blends, the fibre diameter was decreased by increasing the CH contents [111]. The various factors affecting the tensile behaviour of electrospun CC materials (single fibres as well as fibrous membrane) have been investigated in detail [44]. Higher tensile strength was observed in case of smaller diameter fibres. In case of electrospun CC membranes, the increase in the ultimate tensile strength was observed by decreasing CH content [44].

Tan et al. fabricated CC composite materials of variable proportions and evaluated the cell viability and proliferation using cells from a human hemopoietic cell line (K562) [112]. The addition of CH (up to 50%) altered the crosslinking pattern of collagen and cellular proliferation. Further increase in the CH content was linked to reduced porosity and cellular proliferation capacity of scaffolds. In addition, CH improved the physical properties such as better stability of fibrous structure and resisting deformation [112]. The behaviours of CC composite materials were also investigated using human periodontal ligament (PDL) cells. The overall adherence and growth capability of periodontal cells was better while using CC scaffolds compared to collagen or CH-only scaffolds. In terms of adherence and growth of cultured PDL cells, the CC scaffolds were better than either CH or collagen scaffolds. It can therefore be suggested that CC composite scaffolds are promising candidates for PDL tissue regeneration [113]. Considering results of using CC composites for TE applications, a number of investigators attempted electrospinning of collagen CH blends [44,101].

In order to fabricate biocompatible wound healing dressings for promoting regeneration of dermal and epidermal layers; nanocomposite fibrous membranes using collagen and CH were electrospun [114]. Characterization of these materials revealed an appreciable degree of crosslinking that resulted in improved mechanical properties (elastic modulus, strength) coupled with a decreased water sorption capability. In terms of biological properties for specific applications; these electrospun membranes were biocompatible and non-toxic to fibroblasts and promoted wound healing. Considering the wound healing potential, the authors declared these electrospun nano-fibre membranes superior to collagen sponge and gauze [114]. Chen and coworkers [101] electrospun CC blends for TE applications and characterized physical, mechanical, thermal properties and biocompatibility using endothelial and smooth muscle cells. The CC scaffolds showed excellent biocompatibility and proliferation for both endothelial and smooth muscle cells suggesting CC as a promising scaffold material for TE applications for regeneration of nerves and blood vessels [101]. Another study [115] has reported the fabrication of electrospun CC targeting the regeneration of nerves and blood vessels. In addition to natural polymers, synthetic polymers (such as thermoplastic PU) were added to enhance the mechanical properties of TE scaffolds and mimic extracellular matrices. The authors reported promising results of in vitro experiments and suggested that in vivo studies are required to validate these results for vascular and nerve regeneration [115].

A potential application of electrospun CC nanofibrous membranes is for corneal TE [116]. The composite CC membranes showed better optical and mechanical properties compared to CH alone. The addition of collagen has been reported to improve the mechanical and physical properties (such as hydrophilicity, optical clarity) without compromising biocompatibility. In addition, CC membranes showed promising characteristics to promote the attachment, spread and viability of cells and has been suggested as a potential candidate for corneal tissue regeneration applications [116]. Recently, CC electrospun scaffolds have been evaluated for guided bone regeneration applications [117]. The CC nanofibre membranes resulted in enhanced cellular proliferation and expression of osteogenic genes in mesenchymal stem cells. There were no apparent signs of inflammation or tissue reaction in the vicinity of the CC membranes suggesting good biocompatibility and potential for guided bone regeneration applications [117]. Although these materials have been evaluated for a range of TE applications such as skin, nerves, vessels, periodontal and bone regeneration, more in vivo and clinical trials are required to validate their properties prior to clinical applications.

### 3.4. Agarose Chitosan

Agarose is another natural polysaccharide that has been widely used for pharmaceutical and cosmetics applications [118]. Chemically, agarose is a linear polymer composed of repeating units of disaccharide agarobiose. In recent years, many researchers have attempted to fabricate nano-fibres of agarose and CH using various solvents and techniques [119,120,121]. The mixture of trifluoroacetic acid (TFA) and dichloromethane (DCM) has been reported as a suitable ES solvent for agarose and CH. The agarose added to CH lowered the viscosity of the dope remarkably and resulted in a better compatibility and interaction between agarose and CS molecules [120].

The agarose/CH electrospun fibres (from 30–50% agarose) resulted in smooth cylindrical nano-fibres. However, increasing the agarose content further reduces the viscosity leading to relatively thinner fibres and bead formation [120]. The ES of pure CH results in thicker fibres (in the range of few hundred nanometers to microns), hence agarose can be incorporated to reduce and control the average fibre diameter [120]. Besides chitosan, agarose has been blended with other polymers such as polyacrylamide [119] and polyacrylic acid for ES [122].

There are no biocompatibility or cytotoxicity issues for using agarose-CH blends for biomedical applications. Miguel et al. fabricated agarose-CH thermos-responsive hydrogels for wound healing and skin regeneration [123]. The minimal tissue inflammation and improved healing addressed the excellent biocompatibility and supported cellular proliferation [123].

### 3.5. Chitosan PEO Composite Electrospinning

The first documented study focusing on the production of electrospun CH-PEO was conducted by Duan et al. [26] using a combination of various ES parameters [concentration (2–8%), CH-PEO ratio (5:1, 2:1 and 1:1) in 2 wt.% acetic acid], voltage of 15 kV, a flow rate of 0.1 mL/h and a stationary collector. While 2% solution only produced beads with no significant fibre formation and 8% solution was too viscous to produce fibres, optimal results were obtained with solutions containing CH and PEO in the ratios of 2:1 and 1:1. One problematic observation of this study was the inconsistent thickness of the fibre diameter. It was seen that thick fibres in the micrometer range were collected directly under the capillary and thinner fibres in the nanometer range were collected elsewhere. This can be attributed to the repulsion of the CH fibres due to their cationic nature. Increasing the molecular mass of PEO had little effect on the ES capabilities of the solution and fibre morphology [26]. Subsequent studies have aimed to improve the ES characteristics of CH-PEO solutions and achieve better control over the quality and fineness of fibres [124,125,126]. Bhattarai et al. [127] observed that 2% CH and 3% PEO [CH to PEO ratios of 90:10] dissolved in 0.5 M acetic acid produced aligned nano-fibres in the range of 40 nm to 50 µm and claimed improved results compared to Duan et al. [26]. Furthermore, dimethyl sulfoxide was added as a co-solvent to the solution before ES to ease the chain entanglements of CH. An additional advantage of a low PEO is the low swelling of the fibres which increases the structural integrity of the scaffold while in water [127]. In vitro analysis showed that nano-sized CH-PEO fibres favoured cellular attachment and proliferation.

Klossner et al. observed that increasing the total CH-PEO concentration decreases bead formation; however, highly viscous solutions cannot be electrospun [59]. Bead formation was also reduced by decreasing the acetic acid concentration [116]. Additionally, increasing the CH concentration resulted in thicker fibres. Moreover, decreasing the Mw of CH increased the ease of ES. Klossner et al. observed that there was phase separation between CH and PEO after 24 h, hence inhibiting ES [59]. It can be assumed that the CH-PEO solutions in acetic acid have a very short shelf life (<24 h) and must be electrospun within 24 h. The ES capabilities of CH-PEO solutions can be increased by ultra-high molecular weight PEO (UHMWPEO) (Figure 4) [59].

**Figure 5:**
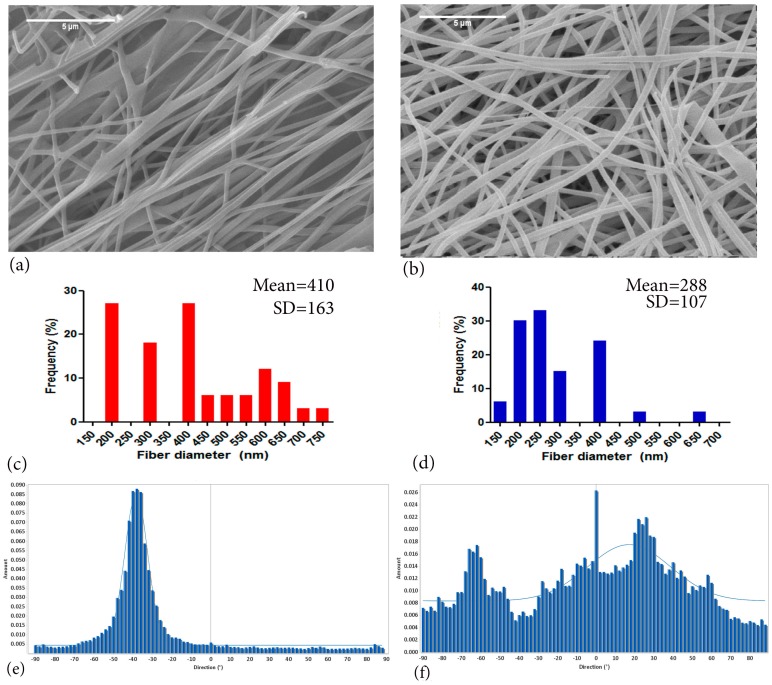
SEM images of electrospun CH-PEO blends; 4.5 wt.% CH:PEO 95:5, and 10:1, 3 wt.% acetic acid in Dimethyl Sulphoxide (DMSO), chitosan electrospun fibres spun using PEO as co-polymer. (**a**) Overly aligned fibres (**b**) random fibres (**c**,**d**) fibre distribution frequency calculated from 100 fibres (**e**,**f**) orientation histograms showing distribution of aligned and random fibres. Image adapted with permission from publisher (scale bar = 5 µm) [3].

Recently, Qasim et al. reported on processing of ES CH-UHMWPEO solutions that contain UHMWPEO as low as 5 wt.% [3,126] (Figure 5). The main advantage of these fibres is the high CH and lower PEO content that can lead to lesser swelling upon immersion in water. Furthermore, increasing the CH proportion can yield enhanced benefits in terms of antibacterial and osseo-conductive properties. Another way of producing CH and PEO is using coaxial ES of two different blends of polymer solutions [128,129]. Ojha et al. electrospun PEO-coated CH fibres that can be exposed by washing away water-soluble PEO [129]. Conversely, Pakarvan et al. have produced similar fibres albeit in the opposite arrangement i.e., a PEO core coated by CH sheath [128]. Upon washing away the inner core of PEO with water, hollow CH nano-fibres were obtained. Hollow fibres can facilitate cellular attachments and proliferation by providing a larger surface area. The potential area of applications may include haemodialysis and wound-dressings.

Plenty of research has been conducted to improve antimicrobial, regenerative properties and stability of electrospun CH-PEO fibres in combination with poly(hexamethylenebiguanide) hydrochloride (PHMB) or silver nitrate nanoparticles to induce antibacterial properties against *Staphylococcus aureus* and *Escherichia coli* [124,130]. These scaffolds can be advantageous for wound dressings for preventing infections and accelerating the healing. In order to improve the surface properties of the fibres, arginylglycylaspartic acid (RGD) peptides can be crosslinked to the fibres via poly(ethylene glycol) following ES [131]. Compared to unmodified CH-PEO fibres, RGD-modified fibres have superior bioactivity and lead to accelerated tissue regeneration. Recently, incorporation of graphene oxide as a carrier for doxorubicin, an anti-cancer drug, to CH-PEO fibres has made these scaffolds useful as a drug delivery medium to target cancerous tissues directly rather than systemic delivery and avoiding numerous adverse effects [132].

**Figure 6:**
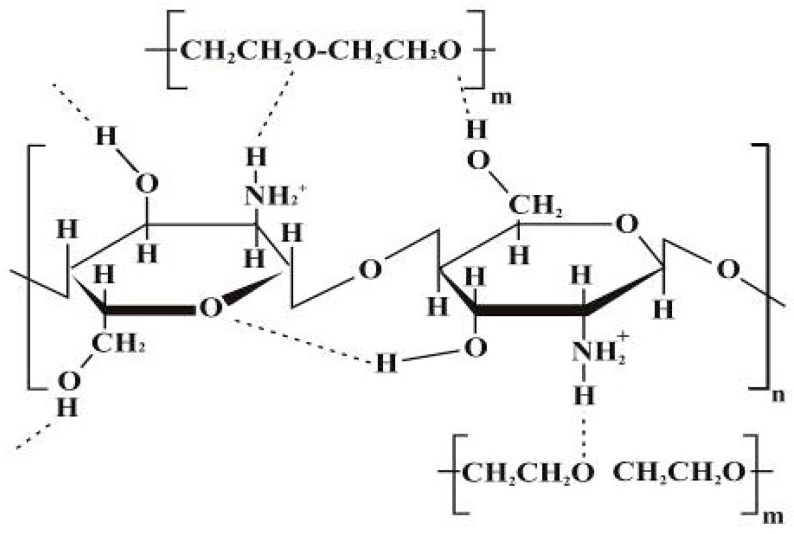
Proposed hydrogen bonding interactions of PEO and CH [125]; (Adapted with permission of publisher).

The PEO (as a copolymer) led to the disruption of the CH chain self-association due to hydrogen bonding between ^−^OH and ^+^H ions originating from water molecules [133]. Subsequently, it diminishes repulsion between CH polycationic groups and triggers chain entanglements to cause fibre formation [124,127]. Pakravan et al. used PEO in different percentages and observed the absorption peak at 1112 cm^−1^ in FTIR spectroscopic analysis [125]. This can be assigned to the ether band shifting to a lower wavenumber. Furthermore, PEO reduces the viscosity by breaching intra and/or intermolecular interactions of CH chains. In addition, the flexible PEO chains form around the rigid CH structures [125]. The interaction of CH-PEO is established as a result of solid hydrogen bonding among OH, CH amino groups (Figure 6) and PEO ether groups [125].

Another way of further improving on the regenerative and mechanical properties of CH-PEO nano-fibrous scaffolds is adding another natural or synthetic polymer such as collagen or poly (ε-Caprolactone) (PCL) to the ES solution. However, production of such scaffolds usually involves the use of chemicals or cross-linking agents such as glutaraldehyde and 1,6-diisocyanatohexane (HMDI) that may cause concern for use in clinical settings. Sarkar et al. used tripolyphosphate (TPP), a cross-linking agent, to successfully produce biocompatible cross-linked CH-PEO (5:1 ratio) fibres having diameters as small as 50 nm in 15 M acetic acid [134]. TPP has previously been used to produce biocompatible and non-toxic CH beads for drug delivery applications [135]. It can be a viable alternative to potentially toxic cross-linking agents such as gluteraldehyde and HMDI.

## 4. Tissue Engineering and Regenerative Applications of Chitosan-Based Solution Electrospun Fibres

### 4.1. Neural Tissue Regeneration

Amongst the available methods for scaffold fabrication, biomedical engineers have utilized electrospinning to aid nerve regeneration by synthesizing nerve guidance conduits or other non-porous templates [136]. The electrospun CH scaffolds have also been studied for their neural regenerative potential. Prabhakaran et al. have shown that rats Schwann cells cultured on PCL/CH fibres exhibit significantly higher biocompatibility compared to PCL fibres [137]. Another exciting prospect in neural tissue regeneration is the possibility of constructing electrospun fibrous nanotubes. Electrospun fibrous collagen/CH/thermoplastic polyurethane nanotubes have shown promising results for cultured Schwann cells [115]. Similarly, in vivo studies conducted on sciatic nerve defects in rats suggested that composite nanotubes consisting of electrospun CH/PVA fibres could function as scaffolds [138]. Additionally, the CH/PVA have superior mechanical properties compared to PVA scaffolds [139]. Generally, electrospun CH fibres having aligned morphology induce higher Schwann cell proliferation compared to random fibres [138].

Although using CH composite scaffolds for neural tissue regeneration seems promising, little is known about their long-term in vivo inflammatory effects. Recently, an in vivo study conducted on multi-layered 3D CH fibres enclosed by a PCL shell has exhibited extensive foreign body reactions while implanted in nerve defects [140]. Hence, more studies are pertinent to develop scaffolds that are considered safe for use in human subjects before they are employed in surgical practice.

### 4.2. Bone Regeneration

Perhaps CH fibres have been most extensively studied as scaffolds for bone regeneration. A typical periodontal defect involves irreversible resorption of alveolar bone. Electrospun CH/PEO scaffolds not only exhibit higher biocompatibility than cast CH membranes, but also possess superior mechanical properties (Table 2) [141].

**Figure 7:**
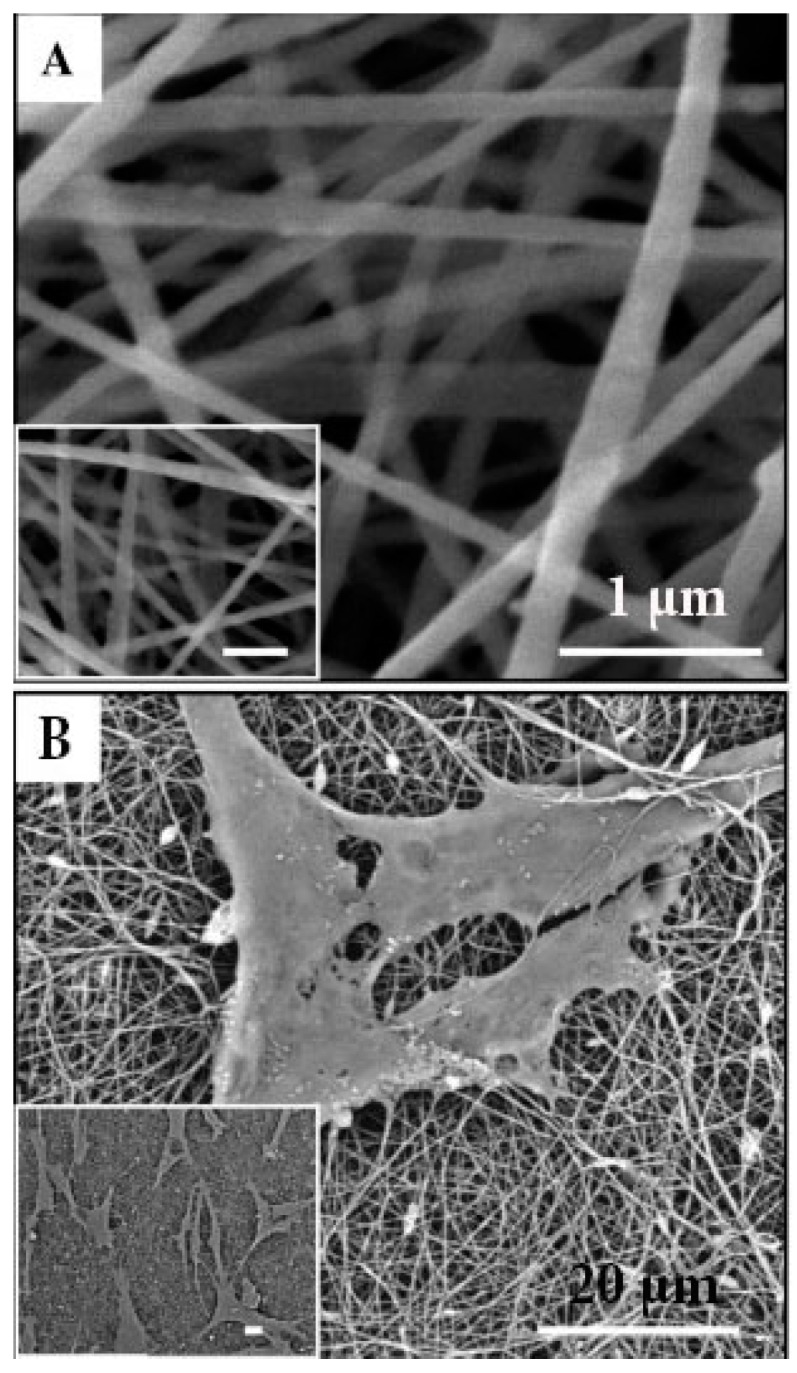
(**A**) SEM micrographs of nanohydroxyapatite/collagen/chitosan fibres; scale bar: 1 µm (**B**) Induced pluripotent stem-cell-derived mesenchymal stem cells (iPSC-MSCs) seeded on HA/chitosan fibres after culturing for 4 days, scale bar: 20 µm (Xie et al., 2016) [142]; (Adapted with permission of publisher).

Bioactive ceramics such as HAp can also be incorporated in CH solutions prior to ES to produce bioactive scaffolds capable of accelerating osteoblast proliferation and bone formation (Figure 7 [152,153]). Various combinations of CH with natural polymers such as silk fibroin, collagen and chitin have also been found to induce accelerated proliferation of osteoblasts and mesenchymal cells in vitro [99,153,154]. Recently, in vivo studies conducted on CC fibrous membranes implanted in bone defects in rabbits have exhibited similar efficacy to commercially available collagen-guided tissue regeneration (GTR) membranes [117].

**Figure 8:**
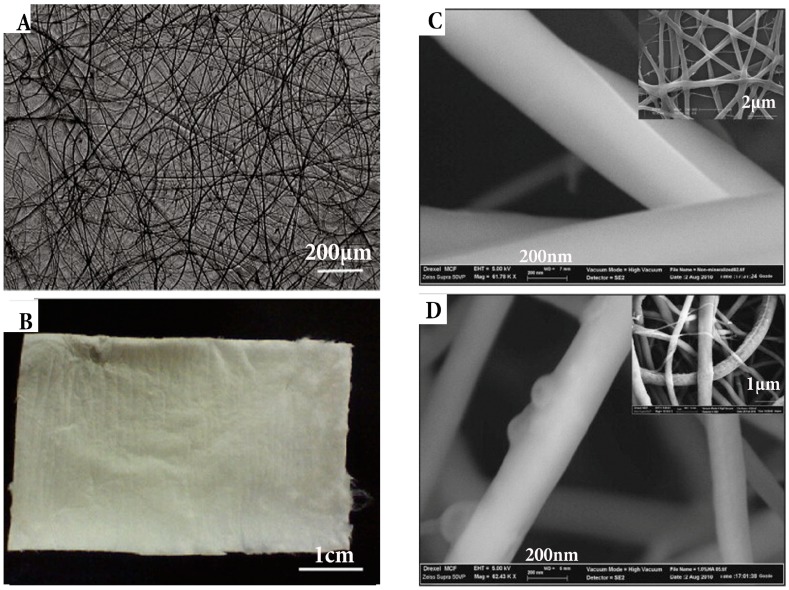
(**A**) Macroscopic image of chitosan fibre and (**B**) fibrous mat; (**C**) Morphology of fibre evaluated by SEM and atomic force microscopy of 0.1% genipin crosslinked and 1% HA loaded; (**D**) 7% chitosan fibres, typical morphology seen inset images [143]; (Adapted with permission of publisher).

As discussed earlier, the mechanical properties and controlled degradation of GTR membranes have been a concern that can be overcome by adding certain non-toxic cross-linking agents such as genipin (Figure 8). Genipin is a natural cross-linking agent that can be used to reinforce CH and extend the degradation period up to 4–6 months as needed for complete bone healing [155]. Mechanical testing has revealed improved ultimate tensile strength (32 MPa) of such scaffolds that is substantially higher than currently available GTR membranes [156,157].

Recent research has focused on developing CH-based GTR scaffolds that can concurrently be used for bone regeneration and drug delivery to the implantation site. Ferrand et al. reported the possibility to immobilize bone morphogenic protein-2 (BMP-2) on electrospun PCL/CH fibrous scaffolds and enhancing the bone regeneration in vivo [158]. More recently, BMP-7 immobilized on PCL-CH fibres has shown superior osteogenic potential compared to fibres without any growth factors when human mesenchymal stem cells (hMSCs) were cultured [159]. Incorporation of growth factors into CH scaffolds have made it possible to ‘kick-start’ bone regeneration rather than function solely as barrier membranes. Coupled with the inherent osteogenic potential of CH, such scaffolds are likely to offer an excellent alternative to conventional GTR membranes.

### 4.3. Drug Delivery

Although CH is primarily used as quaternized form to deliver drugs to the implantation sites, the use of fibrous CH scaffolds as delivery media for various drugs has also been reported [160,161,162]. For instance, electrospun PCL/CH fibres can be used to deliver growth factors for bone regeneration [158,159]. CH fibrous mats impregnated with heparin-bound fibroblast growth factor-2 (FGF-2) stimulated cellular activities of sheep mesenchymal cells indicating a possible mechanism to deliver drugs [163]. Gentamicin immobilized on liposome can be released from CH fibres and has exhibited antimicrobial activity for up to 24 h against *Escherichia coli*, *Pseudomonas aeruginosa* and *Staphylococcus aureus* [164] indicating its potential for wound healing applications. Carbon-based drug carriers such as nano graphene-oxide have also been electrospun along with CH-PEO to produce scaffolds that can release doxorubicin. The primary amino group of CH facilitates cross-linking and ligand attachment for targeted drug delivery. Nanoparticles are negatively charged, and CH is cationic hence promoting electrostatic interaction [132].

### 4.4. Wound Dressings

Considering the excellent porosity and drug-carrying ability of CH fibres, another major application is production of wound dressings [165]. CH can be electrospun along with synthetic and natural polymers such as PVA, silk fibroin and PLLA to produce dressings [100,165,166]. Antimicrobial enzyme lysozyme can be added to CH-PVA fibrous membranes to prevent wound infections [167]. In addition to drugs, nanoparticles can also be co-electrospun with CH. A dual layered membrane of electrospun CH and adipose-derived human extracellular membrane containing nano-titania (TiO_2_) particles exhibits higher healing properties in rats [167]. Similarly, nano-silver particles incorporated into electrospun CH/PEO fibres exhibited antibacterial activity against *S. aureus* and *E. coli*, which are both organisms implicated in wound infections [168]. More recently, electrospun CH/arginine fibres exhibited faster wound healing and anti-bacterial properties [169]. Moreover, CH-PVA fibres containing mafenide acetate have shown antibacterial activity against *S. aureus* and *P. aeruginosa* [170].

### 4.5. Anti-Carious Mucoadhesive Mats

Recently, anti-carious mats constructed from electrospun CH fibres containing antimicrobial agents have been studied for anti-cariogenic potential. CH/thiolated CH mats blended with PVA can be used to deliver anti-caries agents such as *Garcinia mangostana* extract in form of mucoadhesive mats which can be used by patients who may be unable to administer conventional oral hygiene measures to prevent dental caries [171,172].

### 4.6. Other Applications

The diversity of electrospun CH fibres have led to their use as templates for hepatocyte, chondrogenic and myogenic differentiation. Feng et al. reported CH nano-fibre mesh liver TE applications and tested the biocompatibility using hepatocytes [173]. In another study by Noriega et al. CH nano-fibres were used for culturing chondrocytes. Reported results were suggestive that the matrix geometry was able to regulate and promote the retention of the chondrocyte genotype [174]. A number of investigations have been conducted to study cellular interactions and stem cell fate [147,175,176]. Newman et al. studied the effect of topography by synthesizing aligned and randomly oriented fibres on cell shape and cell differentiation towards osteogenic and myogenic lineages [177]. 

## 5. Conclusions and Future Aspects

The present review shows that there is a wealth of scientific evidence available in support of the favourable properties and biocompatibility of chitosan electrospun composite biomaterials for a range of TE and regenerative medicine applications. However, further research including in vivo studies are required to translate these materials from laboratory to clinical applications. Although investigators have been able to alter the instrumentation and solution parameters to mimic natural tissue structure and morphology, the continual process of reporting various possibilities needs further characterisation and clinical trials before their applications for treating medical diseases with predictability. Using electrospinning and augmenting this technique with additives, manufacturers can have further control of the final template. Moreover, clinicians and bioengineers, whilst working together, can solve unexplored regenerative therapies by harnessing the fibre diameter, size, morphology and orientations according to the desired clinical applications. Mimicking structural and functional aspects of natural tissues will have a significant impact on the future of electrospinning of these materials.

## Figures and Tables

**Table 1:** Studies reporting ES of chitosan and silk fibroin composite materials for tissue regeneration application.

Researcher	Solvent	Materials	Key Findings and Significance
Park et al., 2004 [93]	Formic acid	CH/SF blends of variable proportions	Reported the ES of CH/SF blended nano-fibres. The average fibre diameter was reduced with a narrow diameter distribution compared to SF nano-fibres.
Park 2006 [98]	HFIP	Chitin/SF blends of variable proportions	Chitin/SF remains immiscible in nano-fibres The average diameters decreased by increasing chitin contents. Biocompatible and good response for cell attachment and spreading, hence suitable for tissue regeneration applications
Yoo et al., 2008 [94]	HFIP	Chitin/SF blends of variable proportions chitin (5 wt.% in HFIP) and SF (7 wt.% in HFIP)	Confirmed all findings reported by Park et al., 2006 [76]. Chitin/SF solutions were electrospun simultaneously using a hybrid ES technique and nano-fibres were collected on a rotating target. Chitin/SF proportion was controlled by adjusting the flow rates. A narrow fibre diameter distribution (340–920 nm) was observed for chitin/SF nano-fibres compared to SF fibres (140–1260 nm).
Cai 2010 [100]	HFIP, TFE	CH/SF blends; CH contents (0%, 20%, 50%, and 80%)	CH/SF nano-fibrous membranes were successfully electrospun. The average fibre diameter was decreased with the increasing percentage of chitosan. CH/SF composites have better mechanical properties than CS. Electrospun materials were characterized for biocompatibility and antibacterial activity. Authors suggested these membranes as a promising candidate for wound healing applications.
Chen et al., 2012 [101]	mixed solvent [TFA], dichloromethane	CH/SF blends; CH contents (0%, 25%, 50%, 75% and 100%)	Electrospun bead-free CH/SF nano-fibres The composite nano-fibres supported the growth and differentiation of human foetal osteoblasts. Authors reported that a suitable composition of these materials is suitable for bone TE applications.
Zhou et al., 2013 [92]	water	ES dope contained 2.5% (*w*/*v*) CH 9% (*w*/*v*) PVA in an aqueous solution. SF nanoparticles (4–8 wt.%) were added	Electrospun composite nanofibre membranes using water-soluble *N*-carboxyethyl CH/PVA/SF nanoparticles The morphology and diameter of the nano-fibres were affected by silk fibroin nanoparticles contents. Presence of intermolecular hydrogen bonding among the molecules of carboxyethyl CH, SF and PVA. Electrospun nanomaterials demonstrated good biocompatibility and can be considered for potential tissue regeneration applications such as skin regeneration wound dressings.

CH (chitosan); SF (silk fibroin); HFIP (1,1,1,3,3,3-hexafluoro-2-propanol); TFE (2,2,2-trifluoroethanol); TFA (trifluoroacetic acid) dichloromethane; PVA (polyvinyl alcohol).

**Table 2:** Studies conducted on CH and PEO reporting the orientation, mechanical properties, fibre diameters and aiming at clinical tissue engineering (TE) applications.

Application	Solution (Ratio, %)	Fibre Diameter	Young’s Modulus	Orientation	References
Wound dressing	CH:PEO: 0.5 M ACa Triton X or DMSO, 60/40 90/10	few micron down to 40 nm	N/A	Aligned/random	Bhattarai et al. [127]
	4–6 wt.% CH:PEO (2:1, 1:1)	80 to 180 nm	N/A	Random	Duan et al. [26]
	HA/CH (30:70, *w*/*w*) 3 wt.% ACa:DMSO 10:1, 15 wt.% Col, 15 wt.% PEO	190 to 230 nm	N/A	Random	Xie et al. [142] (Figure 7)
	7 wt.% CH: TFA: nHA (0.8%, 1%, 2%)	227 nm ± 154 nm 335 nm ± 119 nm (after CRX Genipin)	142 Mpa ± 13 MPa	Random	Frohbergh et al. [143] (Figure 8)
	CH:PEO (3 wt.% ACa, DMSO, 10:1) UHMWPEO, 5%, 10%, 20%)	114 nm ± 19 nm 138 nm ± 15 nm 102 nm ± 1 nm	N/A	Aligned, Random	Zhang et al. [126]
Skin TE	CH grafted PCL, 25 wt.% PCL (DMF, CLF)	423 to 575 nm	N/A	Random	Chen et al. [144]
	CH/PCL/GEL	890 nm ± 364 nm	N/A	Random	Gomes et al. [145]
	CH/PEO/Henna extract (3/4 wt.%)	89 to 64 nm		Random	Yousefi et al. [146]
Nerve TE	5 wt.% CH: TFA, 10 wt.% PCL (40:60, CH:PCL)	175.82 55.95 (A) 215.79 nm ± 44.2 nm	51.54 MPa (A) 8.85 MPA (R)	Aligned/Random	Cooper et al. [147]
	CH:PEO, 4 wt.% in 50 wt.% ACa (50:50, 70:30, 80:20, 90:10)	60–120 nm	N/A	Random	Pakarwan et al. [41]
	CH:PEO, 1.6% (50 to 90% ACa)	10–240 nm	N/a	Random	Kriegel et al. [133,148]
	CH:PEO, 90% ACa	80 nm ± 35 nm	N/A	Random	Desai et al. [149]
	Ag: 5 wt.% CH:PEO 2 wt.% ACa	100 nm (Ag:CH:PEO) 5 nm (CH:PEO)	(YM) 59.2 ± 22.9 (CH:PEO) 322 ± 36.2 (CH:PEO:Ag)	Random	An et al. [150]
Cartilage tissue regeneration	10 mL of 1% CH sol with x mL 5% PEO	NA	2.25 MPa (YM)	Aligned	Subramanian et al. [139]
	CH (PEO):PCL: HAp (15 wt.%)	200 nm	215 MPa (YM)	Aligned & Random	Wu et al. [151]
Periodontal tissue regeneration	CH:PEO (95:5)	410 nm (A) 288 nm (R)	(YM) 357 ± 136 (A) 259 ± 192 (R)	Random & Aligned	Qasim et al. [3]

(N/A) Not applicable, (TE) Tissue engineering, (YM) Young’s Modulus, (A) Aligned, (R) Random, (CRX) Cross-linking.

## Abbreviations

CH	Chitosan
DMSO	Dimethyl Sulphoxide
Hap	Hydroxyapatite
ES	Electrospinning
nm	Nanometres
PLA	Poly lactic acid
PLGA	Poly lactide co-glycolide
PU	Polyurethane
PS	Polystyrene
PVA	Poly vinyl alcohol
SF	Silk fibroin
TE	Tissue Engineering
TFA	Triflouro acetic acid
PEO	Polyethylene oxide
TPP	Tripolyphosphate
CC	Collagen chitosan
